# Deployment of Solid-Supported Natural Deep Eutectic Solvents via Unmanned Aerial Vehicles to Preconcentrate Contaminants from Environmental Water Samples

**DOI:** 10.1021/acsomega.5c06815

**Published:** 2025-10-06

**Authors:** Vagner Bezerra dos Santos, Carlos D. Garcia, Helayne S. de Sousa, Vinicius A. Carvalho, Severino Carlos Oliveira, Willian Toito Suarez

**Affiliations:** a LIA3 - Applied Analytical Instrumentation Laboratory, Department of Fundamental Chemistry, 28116 Federal University of Pernambuco, Av. Journalist Anibal Fernandes, S/N, University City, Recife, PE 50740-560, Brazil; b Department of Chemistry, 2545 Clemson University, 211 S. Palmetto Blvd, Hunter Rm. 235, Clemson, South Carolina 29634, United States; c Department of Chemistry, 67744 Federal Rural University of Pernambuco, Dom Manuel de Medeiros Street, S/N, Two Brothers, Recife, PE 52171-900, Brazil; d Department of Chemistry, 28120 Federal University of Viçosa, Av. Peter Henry Rolfs S/N, Viçosa, MG 36570-000, Brazil

## Abstract

A simple analytical methodology for extraction and electroanalytical detection of 2-nitrophenol in water is herein presented. The method is based on the preconcentration of the model contaminant on a solid support (sponge) soaked in hydrophobic natural deep eutectic solvents (decanoic acid and menthol) and deployed using an adapted unmanned aerial vehicle. The subsequent analysis was performed electrochemically via square wave voltammetry and boron-doped diamond electrodes. Under optimized conditions, the proposed approach required dipping the sponge impregnated with a hydrophobic NADES in the water sample for only 5 min (well under the autonomy of the drone) and led to a linear range of 0.0250–35.0 μmol L^–1^ and a limit of detection of 2.8 nmol L^–1^. Competitive recovery assays (99 ± 5%) as well as intraday (1.62%) and interday (2.60%) were also obtained, supporting the feasibility of the proposed analytical approach. Moreover, the interferent assays were performed using cations and anions commonly found in environmental waters, which showed no significant effects.

## Graphical abstract

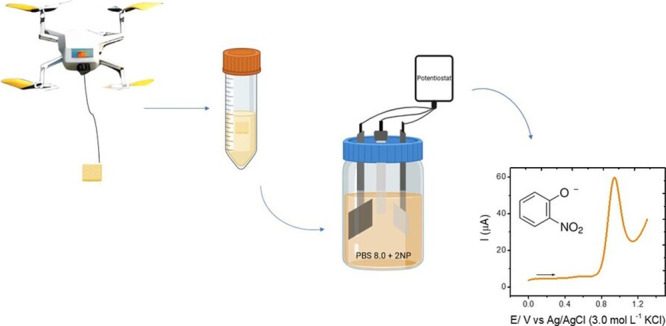

## 1 Introduction

Environmental pollution is regarded as a global problem, posing major challenges for science and technology. Besides traditional targets,^1^ compounds of emerging environmental interest are personal care products, polyfluoroalkyl substances (PFAS), endocrine disruptors, and a wide range of pharmaceutical compounds.^2,3^ Due to their environmental impact direct effects on human health,^3^ many of these compounds can only be removed from wastewater using specific processes.^4–6^ Among those, nitrophenols are particularly important not only because of their toxicity, notable stability, and water solubility^7^ but also because they can be released from a variety of processes, including combustion of fossil fuels, manufacturing practices, and agricultural operations.^8^ Nitrophenols are also degradation products of other contaminants, thus providing the opportunity to use them as model compounds for the development of remediation strategies.^9,10^ Since the United States Environmental Protection Agency (US-EPA) has classified nitrophenols as priority pollutants, their concentrations in water samples are tightly monitored,^11^ noting that aquatic organisms can be significantly affected when 2-NP > 73 μg·L^–1^ (0.52 μmol L^–1^)^12^ and that concentrations of 2-NP as low as 230 μg·L^–1^ (1.65 μmol L^–1^) can lead to acute toxicity.^13^ These concerns have fostered the development of a wide range of analytical technologies^14,15^ for the analysis of nitrophenols in water, including those based on high-performance liquid chromatography,^16–18^ gas chromatography,^16^ capillary electrophoresis,^19,20^ or electroanalytical methods.^21–24^ While many of these reports provide reasonable avenues for the quantification of these contaminants, their low concentration ranges and the presence of interferences typically require the integration of a preconcentration step in the analytical workflow. Toward this goal, a wide range of extraction/preconcentration methods have been applied, including solid-phase,^25^ stir-bar adsorptive,^26^ electromembrane,^18^ and various liquid–liquid microextractions.^14,27,28^ Besides their high costs, many of these extraction methods involve the use of large amounts of organic solvents, which cannot be deployed on-site and impair the green metrics of the analysis. In this sense, the use of deep eutectic solvents (DES) can provide a reasonable alternative to address this problem, as they have been extensively used for the extraction of various pollutants^29–31^ (including nitrophenols^32,33^ ) from environmental waters. DES are formed by specific combinations of hydrogen bond donors and acceptors, leading to liquid mixtures with a melting point that is significantly lower than those of the corresponding precursors.^30,34^ Among those, natural deep eutectic solvents (NADES)^35–37^ are a subclass of DES formed by natural compounds that provide similar extraction capacity with the additional advantages of featuring low toxicity, low flammability, and biodegradability.^38–41^ Besides enabling their rational design to address specific needs in terms of polarity or reactivity,^34^ these systems offer several additional advantages^42^ including their low volatility^43^ and the possibility of being prepared on-site.^44^ These aspects are particularly important for the development of safe and effective point-of-need analytical methods for water contaminants, minimizing the need to transport toxic reagents and, thus, avoiding potential incidents. As an example of the potential of NADES to facilitate the analysis of water samples, our group recently described a simple method to quantify surfactants using a modified version of the methylene blue active substances (MBAS) that integrates smartphone detection.^45^ While practical, fast, and achieving an almost perfect greenness score (0.96), the method still required the manual collection of the samples.

Aiming to address this limitation, this report describes a sampling approach for water bodies, where a known volume of NADES is deployed by using an unmanned aerial vehicle (UAV) or drone. The approach represents an extension of previous drone-based methods^46–52^ and is based on the use of a sponge impregnated with a hydrophobic NADES (pH* = 4 ^53^), immersed in the water to be sampled using a fishing rig (hook, line, and weights) attached to the drone. The main advantage of the described methodology, besides those provided by traditional reactive liquid–liquid extraction (LLE),^54,55^ is that it enables sampling large areas (even those distant from the shore) safely and quickly. To the best of our knowledge, this is the first report describing the use of a UAV to perform on-site extraction of pollutants using NADES. As a proof of concept, 2-nitrophenol (2-NP) was used as a model analyte, noting that other nitrophenols feature similar chemical reactivity,^56,57^ potentially enabling the development of subsequent methodologies for the detection of fungicides,^58^ pesticides,^59^ pigments,^60^ or pharmaceuticals.^22,23^

## 2 Materials and Methods

### 2.1 Reagents and Solutions

All reagents used for the experiments were of analytical grade and used as received. All the aqueous solutions were prepared using deionized water (>18.0 MΩ.cm) from a Millipore Milli-Q system (Merck, Saint Louis, USA). Sulfuric acid was purchased from BDH (Sigma-Aldrich, Saint Louis, USA). Decanoic acid, menthol, and 2-nitrophenol (2-NP) were purchased from Sigma-Aldrich (Saint Louis, USA). Phosphate buffer solutions (0.5 mol L^–1^), from pH 2.0 to 10.0, were prepared by mixing phosphoric acid, sodium dihydrogen phosphate, and/or disodium hydrogen phosphate (Sigma, Saint Louis, USA). Other compounds used include potassium hexacyanoferrate (III) (Sigma-Aldrich, Saint Louis, USA), sodium chloride (Bayer, Saint Louis, USA), nickel chloride (Mallinckrodt, Kansas, USA), copper chloride (Sigma-Aldrich, Mallinckrodt, USA, USA), calcium carbonate (Grenrel Storage, Alabama, USA), magnesium sulfate (Mallinckrodt, Kansas, USA), aluminum sulfate (Fisher Science, Pittsburgh, USA), bismuth nitrate (Alfa Aesar, Massachusetts, USA), iron­(III) chloride (Alfa Aesar, Massachusetts, USA), iron­(II) sulfate (Acros Organics, Wisconsin, USA), potassium phosphate (Sigma-Aldrich, Saint Louis, USA), potassium bromide (Alfa Aesar, Massachusetts, USA), and sodium dihydrogen phosphate monohydrate (Fisher Scientific, Pittsburgh, USA). Stock solutions of 2-NP were prepared in deionized water.

### 2.2 Apparatus

Chronoamperometry, cyclic voltammetry, and square wave voltammetry were performed using an Emstat 4.0 potentiostat, controlled using the PSTrace 5.11 software (PalmSens, Indiana, USA). The electrochemical measurements were performed using a miniaturized (6 mL) three-electrode electrochemical cell. This electrochemical cell included a 25 mm^2^ boron-doped diamond electrode (BDDE) (Boromond; Changsha, China), doped with 8000 ppm boron, and used as a working electrode (WE). Additional details related to the assembly of the WE are described elsewhere.^61^ A Ag/AgCl (3.0 mol L^–1^ KCl) electrode was used as the reference electrode (RE), and a 1 cm^2^ platinum electrode was used as the counter electrode (CE). A Thermo Scientific (Pittsburgh, USA) Orion Start A111 pH meter with a Ag/AgCl 3.0 mol L^–1^ KCl glass electrode (9157BNMB) was used to measure the pH of the buffer solutions and water samples. To carry out the surface water sampling, a UAV (Mavic Air manufactured by DJI, New York, USA) was purchased. It is a rather small (168 × 184 × 64 mm) and light 430 g quadcopter that features an autonomy of 21 min. Prior to all experiments, the aircraft’s firmware was updated (if required), and the integrated GPS system was calibrated using at least seven satellites.

### 2.3 NADES Preparation

The NADES were prepared following a similar approach that was recently described by our research group,^45^ using artificial intelligence to predict the formation of NADES with hydrophobic features. Briefly, a Python script was first used to generate random mixtures of hydrogen bond donors (HBD) and hydrogen bond acceptors (HBA) varying in stoichiometric ratios (1–5), number of components (1–5), and chemical structure (*n* = 198). Then, the formation probability (*p*) for each mixture was calculated using the developed algorithm, leading to the selection of a hydrophobic NADES formed with decanoic acid and menthol (1:1 molar ratio). For the preparation, the corresponding amounts of reactants were weighed, mixed, and then heated at 85 °C under stirring until a clear liquid was formed. It is important to note that, albeit predicted by our algorithm to be stable and hydrophobic, NADES formed by various ratios of menthol and decanoic acid display low surface tension,^62^ are mildly hydrophobic,^63,64^ feature an adequate safety profile,^40,65–68^ and have been previously used as extraction media.^41,53,68–70^

### 2.4 NADES Extraction

2-NP (p*K*_a_ = 7.2) was used as a model compound to investigate the feasibility of the proposed approach. Surface water samples were collected from locations surrounding Clemson, SC (Figure S1) either manually (shore) or with the help of the UAV and analyzed via (I) liquid–liquid extraction using only NADES (LLE-NADES), (II) solid-supported liquid–liquid extraction using a paper substrate (paper-NADES), or (III) solid-supported liquid–liquid extraction using a sponge as the support (sponge-NADES). Following a previously reported procedure,^45^ manually collected samples (2 L, filled by immersion from the shore) were placed in brand-new polypropylene bottles, labeled, and then kept at 4 °C until use. For the samples collected with the UAV, three different approaches were investigated. The first customization of the UAV employed a 5 V miniaturized pump (Aliexpress, Zhejiang, China, 38 × 21 mm) that was powered by a high-current driver (ULN2003) connected to a power bank (5 V/1 A, FLIR; Figure S2). This sampling system is simpler and lighter (only 130 g) than the one previously reported by us,^51^ which employed solenoid valves and an Arduino board. The water sampling system featured flow rates of 30 mL/min and was used for the LLE-NADES procedure (case I). For the cases using either the paper or the sponge, the solid supports were immobilized to a nylon fishing line (length: 2.0 m), using a fishing hook, and placed 50 cm above a 25 g fishing weight (used to avoid the pendulum effect due to the wind generated by the rotors). This arrangement (Figure S2) enabled safe flying of the UAV over the target area and lowering of it to immerse the support to a depth of approximately 1 m during a period of 5 min. Like other SS-LLE,^71,72^ the selected sponge only allowed supporting the NADES and provided no interaction with the analyte (data not shown). For each strategy, the effects of both NADES volume and extraction time on the extraction efficiency were preliminarily investigated, leading to the selection of the experimental conditions described in Table[Table tbl1]. It is important to note that besides providing physical durability, the sponge was able to retain a volume of NADES 4 times larger than the paper.

**Table 1:** Summary of the Sample Collection Methods Used in This Method, Along with the Corresponding Experimental Conditions

Method	Sample collection	Solid support	Sample volume	Volume of NADES
LLE-NADES	Manual		10 mL	3.0 mL
LLE-NADES	UAV		10 mL	3.0 mL
Paper-NADES	UAV	250 ± 10 mg		0.5 mL
Sponge-NADES	UAV	100 ± 5 mg		2.0 mL

After the preconcentration step, the NADES (containing 2-NP) were immersed in phosphate buffer (pH = 8.0) to deprotonate the 2-NP and promote the release of 2-NP to the aqueous phase, which was then used for the electrochemical analysis (*vide infra*). It is important to highlight that the selected NADES (δ = 0.91 g·mL^–1^)^53^ formed a stable biphasic system when mixed with water, facilitating its use as LLE media but requiring the use of weights to immerse the sponge or paper used in our experiments.

### 2.5 Electrochemical Analysis

The BDDE surface was first pretreated by sequentially applying 200 mA for 120 s and −200 mA for 480 s (in 0.5 mol L^–1^ H_2_SO_4_). The electrochemical performance of the pretreated BDDE surface was then evaluated via voltammetry using 1.0 mmol L^–1^ [Fe­(CN)_6_]^3–^ solution in 0.1 mol L^–1^ KCl,^46,47,73^ showing the expected reversible redox behavior (data not shown). These electrodes were then used to quantify 2-NP in environmental samples using square wave voltammetry (SWV) by dispensing a known volume of NADES (containing 2-NP) into the electrochemical cell (6 mL of PBS, pH = 8.0) and letting the system equilibrate for 10 min. Optimization of the analytical parameters was performed considering the potential increment (ΔEs, from 1 to 10 mV), amplitude (*a*, 10–100 mV), and frequency (*f*, 10–100 Hz). Under optimized conditions, the corresponding calibration curves were obtained, allowing the determination of the sensitivity, linear range, limit of detection (LOD= 3 σ/slope), and limit of quantification (LOQ= 10 σ/slope). The intraday and interday repeatability, evaluation of potential interferences, and recovery tests were also carried out to verify the precision, robustness, and accuracy of the electroanalytical methods developed. The interday repeatability tests were performed using a solution of 3.89 μmol L^–1^ 2-NP. The study of probable interferents was carried out by a 1:100 (interferent/analyte) ratio containing different cations and anions normally found in environmental waters^2,23,24,46,51^ using the same 2-NP solution. For the recovery tests, aliquots of 2-NP standard solutions at concentrations of 2.89 and 3.89 μmol L^–1^ were added to the environmental water samples. All data were collected at room temperature in triplicate.

## 3 Results and Discussion

### 3.1 Effect of pH on the Electrochemical Response of 2-NP

To gain insights related to the oxidation process of 2-NP on BDDE, preliminary experiments were performed by cyclic voltammetry. As shown inFigure S3, two clearly defined electrochemical processes were observed in the voltammograms, attributed to the irreversible oxidation of the phenolic group (anodic peak, Ep > 0.8 V) and the reduction of the −NO_2_ group (cathodic peak, Ep < −0.5 V), respectively. In line with the reaction mechanism described in previous reports,^74,75^ it was also observed that the oxidation reaction was favored at alkaline conditions while the reduction reaction was enhanced at acidic solutions, leading to a significant dependence of both Ip and Ep with respect to the solution pH. Based on these results and aiming to reach a sensitivity that was relevant for environmental analysis, the next step was an investigate effect of solution pH on the response (Ip and Ep) via SWV. A summary of these results is presented in Figure[Fig fig1]A, where a similar dependence to that obtained by cyclic voltammetry (Figure S3 A) was observed, featuring a maximum in the anodic response at pH = 8.0. Moreover, and also in line with previous reports,^22,24^ our results also showed a significant dependence of the Ep with respect to the solution pH (Figure[Fig fig1]B). Based on these results (higher current values and lower detection potential), the oxidation process at pH = 8.0 was selected as the most adequate for quantification purposes. Under these conditions, the electrochemical oxidation of 2-NP is expected to proceed in a series of multiple steps (to 2-nitrophenol, 2-hydroxylaminophenol, and 2-aminophenol), involving the exchange of 2H^+^,^76^ and providing an adequate balance between electrochemical signal and stability, an issue that affects various carbon electrodes.^77,78^ In addition, and while other groups have reported the possibility to perform the detection in the DES,^79,80^ this pH value is also sufficient to transfer of 2-NP from the NADES phase into an aqueous solution (pH > p*K*_a_) prior to its electrochemical detection.

**Figure 1:**
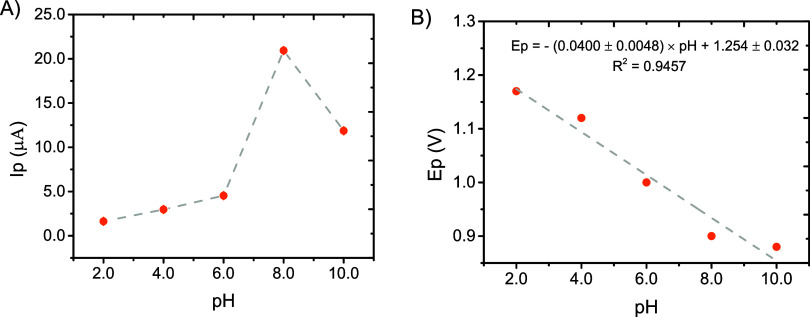
(A) Dependence of the anodic peak current with respect to solution pH, for 2-NP (60 μmol L^–1^) investigated via SWV (ΔEs = 8 mV, *f* = 70 Hz, *a* = 30 mV). All solutions were prepared in 0.5 mol L^–1^ phosphate solutions. (B) Dependence of the anodic peak potential with respect to solution pH, for 2-NP (60 μmol L^–1^).

### 3.2 SWV Optimization and Parameters of the Electrochemical Oxidation of 2-NP

Aiming to maximize the anodic response, the experimental parameters of SWV were optimized, considering the effect of the potential step (ΔEs, 1 to 10 mV), the amplitude (*a*, 10 to 100 mV), and the frequency (*f*, 10 to 100 Hz) on the signal intensity. As it can be observed in Figure S4, a significant dependence of the peak current was obtained as a function of each of the selected variables, leading to the adoption of ΔEs = 8 mV, *a* = 30 mV, and *f* = 70 Hz as the optimum parameters. Under these conditions, the relationship between Ep vs lnf was then investigated to confirm the number of electrons that participate in the electrochemical oxidation of 2-NP.^49,81–83^ *T* is the absolute temperature (293.15 K), α is the electron transfer coefficient (0.5), *n* is the number of electrons, *F* is the Faraday constant (F = 96.485 C mol^–1^), and *R* is the ideal gas constant (*R* = 8.314 J K^–1^ mol^–1^). As shown in Figure S4, the slope obtained was 0.0203, indicating the participation of two electrons in the electrochemical oxidation of 2-NP.^84^ Additionally, considering the shift in peak potential with respect to the solution pH, and the slope of the Nernst’s equation (Figure[Fig fig1]B), it can be deduced that 2 H^+^ also participate in the reaction, as previously noted.^81,84–86^

### 3.3 Reactive Liquid/Liquid Extraction

As described in Section 2.4, the effectiveness of four different strategies to preconcentrate 2-NP from water samples was investigated (Table 1). In each of these cases, the effect of the volume of NADES used and the extraction time was initially assessed using an aqueous sample containing 30 μmol L^–1^ 2-NP. After each preconcentration step, the NADES (containing 2-NP) were then transferred to the electrochemical cell containing PBS (6 mL, 0.5 mol L^–1^, pH 8.0) and analyzed electrochemically via SWV (scanning from 0 to 1.3 V). For the first case (manual LLE using 3.0 mL of NADES, used as a control), it was observed that beyond the initial values considered within the experimental design, the sample volume and extraction time only had a minor effect on the extracted amount, leading to the selection of 10 mL of sample, extracted during 10 min (Figure S5) as the optimum parameters. It was observed that under such conditions, however, the volume of NADES used had a significant effect on the amount of 2-NP being extracted from the sample and then transferred to the buffer solution. In this case, a significant improvement in the signal was obtained in the 10–200 μL. Further increases in the volume of NADES used (up to 800 μL) did not render significant improvements in the detection of 2-NP, leading to the selection of 200 μL as the optimum volume of NADES. Under these conditions, most of the initial 2-NP from the sample was extracted, leading to an enrichment factor of 5X (Figure S5).

The extraction of 2-NP from water samples, using NADES immobilized onto the selected solid supports, was then investigated as a function of sample volume and extraction time. As summarized in Figure[Fig fig2]A, similar trends were obtained for different sample volumes (except for the experiment performed using only 1 mL of sample, where the amount of analyte becomes limiting) and extraction times. In fact, all sample volumes >10 mL rendered comparable results, as evaluated via SWV, measuring the 2-NP recovered in the buffer solution. These results can be attributed to the relative hydrophobic nature of 2-NP (log*P* = 1.77), which favors its partition onto the hydrophobic NADES phase (first) and the subsequent release to the alkaline aqueous buffer. Moreover, this finding is exceptionally convenient for our application, as clearly supports the idea of using the immobilized NADES for direct analysis in water bodies. The analysis of the electrochemical response as a function of the extraction time (Figure[Fig fig2]B) shows that the equilibrium was reached within 10 min, for both the paper- and the sponge-immobilized NADES. This not only is well within the capabilities of the drone (21 min flight autonomy) but also represents a faster alternative to traditional methods, avoiding the need for time-consuming centrifugation processes.^87^ Despite these similarities, it is important to note that the extraction using sponge-NADES rendered a slightly higher signal than the extraction using paper-NADES, leading to a higher sensitivity of the method (Figure S6).

**Figure 2:**
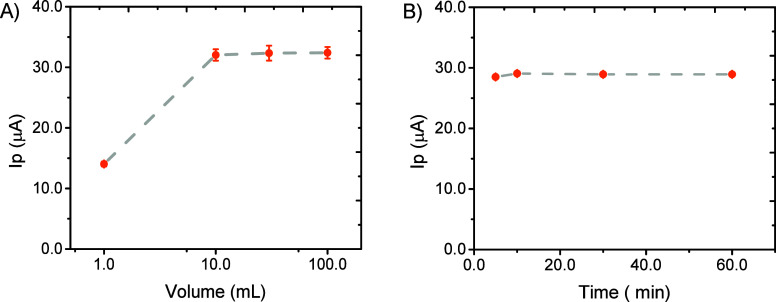
Effect of sample volume (A) and extraction time (B) on the extraction of 2-NP (30.0 μmolL^–1^) from water samples using NADES immobilized on the paper. 2-NP determined via SWV (PBS, pH 8.0, *f* = 70 Hz, ΔEs = 8 mV, *a* = 30 mV). The current using sponge-NADES was 30% higher than the paper-NADES.

It is also important to mention that despite the low toxicity and the hydrophobic nature of the selected NADES,^37,71,72,88^ one of the possible concerns is the possibility to leave residues on the water. Therefore, both the sponge and the paper substrates modified with the NADES were weighted after and before dipping them into a beaker containing 10.0 mL of water. This procedure was carried out 3 times, leading to insignificant mass changes (Figure[Fig fig3]) upon immersion in the water sample. These results along with the observation that no residue was found on the surface of the water after immersing these substrates are in agreement with previous reports^65^ and support the idea of using the selected NADES as a viable option over traditional organic solvents^41,89–92^ to preconcentrate contaminants from water bodies. Furthermore, the utility of a similar DES (menthol:oleic acid) in marine coatings was recently demonstrated by Duarte’s group.^66^

**Figure 3:**
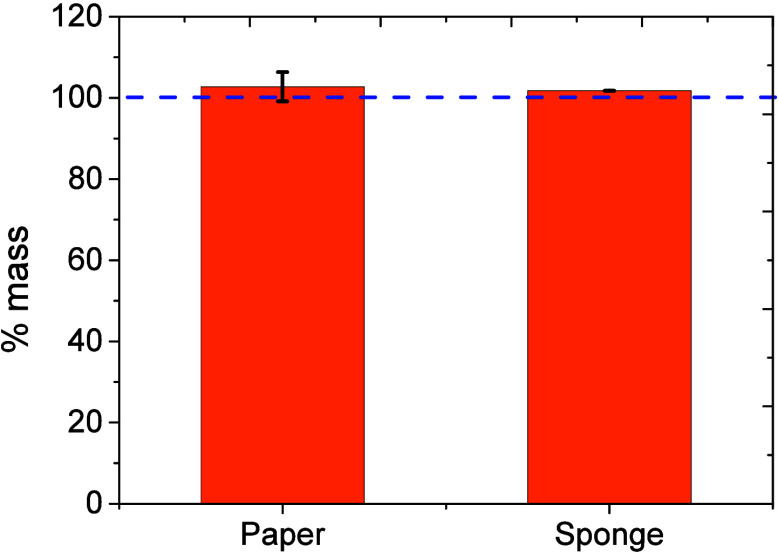
Mass change of the NADES immobilized on either the paper or the sponge before and after immersion in 10.0 mL of water. Data in triplicate.

### 3.4 Quantification of 2-NP Using SWV

After optimizing the SWV parameters (Figure S4), calibration curves were constructed for 2-NP to determine the corresponding figures of merit (linear range, sensitivity, LOD/LOQ, and inter/intraday repeatability). For these experiments, water samples containing known concentrations of 2-NP were prepared, extracted with NADES, recovered in buffer, and then analyzed via SWV using the anodic reaction to generate corresponding peak current values. Representative results, accounting for the full analytical process, are provided in Figure S7. First and foremost, it is important to state that the three selected approaches (LLE-NADES, paper-NADES, and sponge-NADES) enabled the extraction of 2-NP from water samples and subsequent SWV analysis. In all cases, the selected strategies were able to detect 2-NP in concentrations lower than 230 ppb (1.65 μmol L^–1^) and 73 μg L^–1^ (0.52 μmol L^–1^), which are the thresholds set by US-EPA for fresh^13^ and environmental waters,^12^ respectively. Further analysis of these results enabled calculating the corresponding analytical figures of merit for the selected methodologies, which are summarized in Table[Table tbl2]. While our results showed that the most efficient extraction method was sponge-NADES (leading to a sensitivity of 1.017 ± 0.003 μA.L.μmol^–1^), the use of the traditional LLE-NADES extraction provided the widest linear range, potentially enabling its use for highly contaminated sites. In line with these results, the use of the sponge as a matrix to immobilize the NADES also led to competitive LOD/LOQ values^22,25,32,84,93^ and intra/interday variabilities surpassing those reported in recent reports.^55,94,95^

**Table 2:** Analytical Figures of Merit Obtained for the Selected Analytical Methodologies

Method	Linear range (μmol L^–1^)	slope (μA·L·μmol^–1^)	***R*** ^2^	LOD (nmol L^–1^)	LOQ (nmol L^–1^)	Intraday (%)	Interday (%)
**LLE-NADES**	0.058–110	0.152 ± 0.005	0.997	18.8	62.4	0.49%	0.65%
**paper-NADES**	0.058–35	0.759 ± 0.003	0.993	3.95	13.1	1.31%	3.32%
**sponge-NADES**	0.025–35	1.017 ± 0.003	0.992	2.81	9.37	1.62%	2.60%

Moreover, to determine if the methodology using NADES was significantly affected by common interferences, cations and anions normally found in environmental water samples^49,50,96,97^ were studied as potential interferent. The interference study was performed at 1:100 ratios for a concentration of 3.9 μmol L^–1^ (Figure[Fig fig4]). None of the ions studied rendered significant interference for detection of 2-NP using the sponge-NADES approach. This result can be attributed to the low affinity of the hydrophobic NADES for hydrophilic compounds, such as the cations and anions commonly found in environmental waters.^49,50^ Additionally, the SS-LLE is known an extraction method where the interference is reduced, leading to competitive values of recovery with low levels of interference and, thus, enhancing the accuracy of the analytical methodologies.^72^ Based on a brief review of some results reported in the literature,^21–24^ the methodology developed here presented a higher level of automation toward water sampling, with low cost, low waste generation, and green metrics.^48,51,52,97,98^ Moreover, the methods allowed *in situ* electroanalytical detection, offering a limit of detection as low as 2.81 nmol L^–1^, one of the lowest reported for nitrophenols^99–104^ (see Table S1).

**Figure 4:**
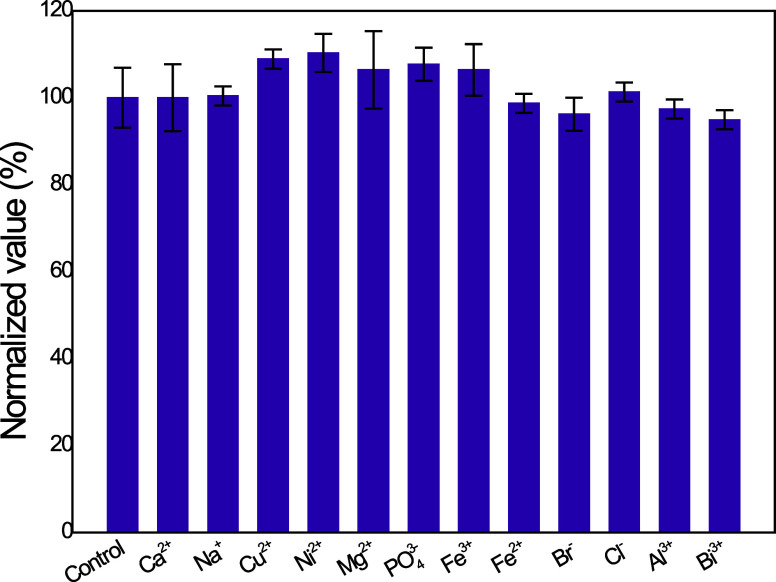
Interference study. The control consisted of a 2.89 μmolL^–1^ 2-NP solution. The interferents studied presented a not significative error for 2-NP analysis with a relative error ranged from −5.10 to 10.3% for a 1:100 (analyte:interferent) ratio.

### 3.5 Environmental Water Samples

To demonstrate the analytical advantages of the proposed methodology, water samples were collected using both automatic and manual methods (as described in Section 2.4). Figure[Fig fig5] shows a sequence of photographs related to the sponge-NADES, where the UAV is shown at the shore, followed by the drone’s view during flight and during the immersion of the sponge. Analyses performed (using optimized conditions) on as-collected samples from the lake did not find 2-NP above the LOD of the developed method. These results were somewhat expected as, despite the well-documented contamination with PCBs,^99^ there are no reports of incidents related to 2-NP.

**Figure 5:**
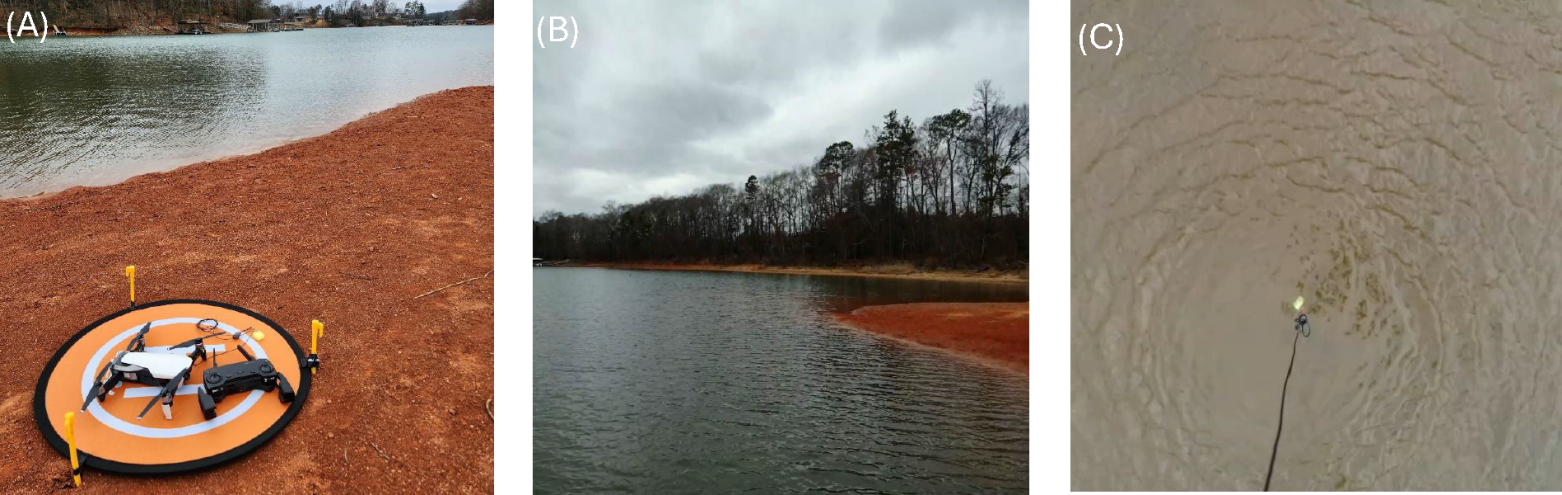
UAV was adapted with sponge-NADES for SS-LLE. UAV aircraft, its control, and the line fishing with sponge coupled to it (A). The image capture to the UAV camera to site to the water collected (B). The sponge-NADES was dipped to collect the possible pollutant (C).

In addition, aliquots of 10.0 mL of environmental water samples collected by the UAV adapted using a micropump were spiked with 2-NP (to obtain concentrations of 2.89 and 3.89 μmol L^–1^) and analyzed to calculate the corresponding recovery values. Results collected for each methodology are provided in the Supporting Information section of the manuscript and show that while the traditional approach (LLE-NADES, see Table S2) may slightly overestimate the concentration of 2-NP in the sample (102 ± 11%), the immobilization of NADES in paper (Table S3, 101 ± 4%) or the sponge (see Table S4, 99 ± 5%) leads to slightly better results, potentially addressing manual sampling inconsistencies and representing an additional advantage of SS-LLE.^72^

## 4 Conclusions

The present report describes the possibility to immobilize NADES in a solid support (sponge), greatly facilitating its use for reactive L-L extraction of water contaminants. In this specific case, the immobilized NADES were deployed using a UAV and a fishing rig, enabling the possibility to obtain samples from a lake. To demonstrate this possibility, hydrophobic NADES (decanoic acid and menthol, 1:1 molar ratio) and 2-nitrophenol were used as model solvents and contaminant, respectively. Our results showed that the use of NADES enabled the preconcentration of 2-NP (5×) and subsequent analysis using SWV and represented, to the best of our knowledge, the first report describing this approach. Out of the investigated possibilities, the use of a common sponge was the most convenient solid phase, supported the largest volume of NADES. This approach led to an LOD of 2.81 nmol L^–1^, one of the lowest LOD values reported thus far. Overall, the proposed approach (sponge-NADES coupled with the use of a UAV) represents one of the most efficient ways to sample large water bodies, reducing the sampling time, increasing access to difficult sites, and greatly reducing the number of professionals involved in the analysis.

## Supplementary Material



## Notes

Map with the sample collection sites; pictures of the drone, adapted to perform the three different water sampling strategies; effect of pH on the electrochemical response; SWV optimization; results of SWV for LLE of 2-NP using NADES; results of SWV for SS-LLE of 2-NP using paper-NADES and sponge-NADES; analytical curves for quantification of 2-NP; comparison of analytical figures of merit for various electrochemical methods; recovery data for the methodology using LLE-NADES; recovery data for the methodology using paper-NADES; and recovery data for the methodology using sponge-NADES (PDF)
