# Supplementary material for: Deployment of Solid-Supported Natural Deep Eutectic Solvents via Unmanned Aerial Vehicles to Preconcentrate Contaminants from Environmental Water Samples
Source: ACS Omega. 2025 Oct 6;10(41):48808–17. doi: 10.1021/acsomega.5c06815 (PMC12547756; doi:10.1021/acsomega.5c06815)
Supplement: Supplementary file 1 [file ao5c06815_si_001.pdf]

Supplementary Information

Deployment of Solid-Supported Natural Deep Eutectic Solvents via Unmanned Aerial Vehicles to Preconcentrate Contaminants from Environmental Water Samples

Vagner Bezerra dos Santos[a,b*], Carlos D. Garcia[b,*], Helayne S. de Sousa[a], Vinicius A. Carvalho[a],

Severino Carlos Oliveira[c] and Willian Toito Suarez[d]

[a]LIA[3] - Applied Analytical Instrumentation Laboratory, Department of Fundamental Chemistry, Federal University of Pernambuco, Recife, 50740-560, PE, Brazil (Av. Journalist Anibal Fernandes, S/N, University City)
[b] Department of Chemistry, Clemson University, Clemson, South Carolina 29634, United States (211 S. Palmetto Blvd, Hunter Rm. 235)
[c]Department of Chemistry, Federal Rural University of Pernambuco, Recife, 52171-900, PE, Brazil
(Dom Manuel de Medeiros Street, S/N, Two Brothers)
[d]Department of Chemistry, Federal University of Viçosa, Viçosa, 36570-000, MG, Brazil (Av. Peter Henry Rolfs S/N)

[*]*Corresponding author:
vagner.bsantos@ufpe.br / Recife, 50740-560, PE, Brazil (Av. Journalist Anibal Fernandes, S/N, University City)
cdgarci@clemson.edu / Clemson, South Carolina 29634, United States (211 S. Palmetto Blvd, Hunter Rm. 235)

Table of Contents

Figure S1: Map with the sample collection sites. 6
Figure S2: Pictures of the drone, adapted to perform the three different water sampling strategies....6
Figure S3 A: Effect of pH on the electrochemical response. 8
Figure S4: SWV optimization.. 9
Figure S5: Results of SWV for LLE of 2-NP using NADES.. 10
Figure S6: Results of SWV for SS-LLE of 2-NP using paper-NADES and sponge-NADES 11
Figure S7: Analytical curves for quantification of 2-NP....12
Table S1: Comparison of analytical figures of merit for various electrochemical methods....13
Table S2: Recovery data for the methodology using LLE-NADES. 14
Table S3: Recovery data for the methodology using paper-NADES. 14
Table S4: Recovery data for the methodology using sponge-NADES. 14

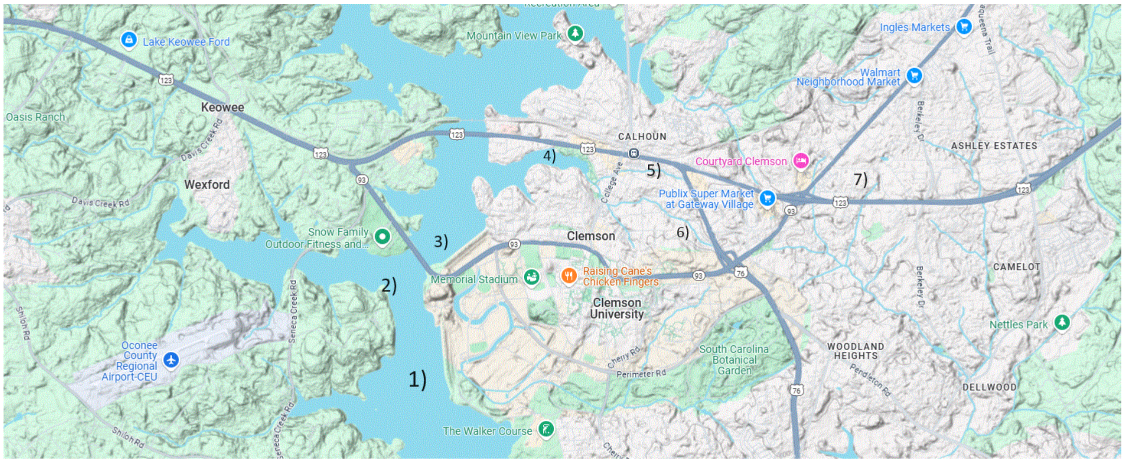

Figure S1: Map with the sample collection sites (from 1 to 7) in Clemson, SC.

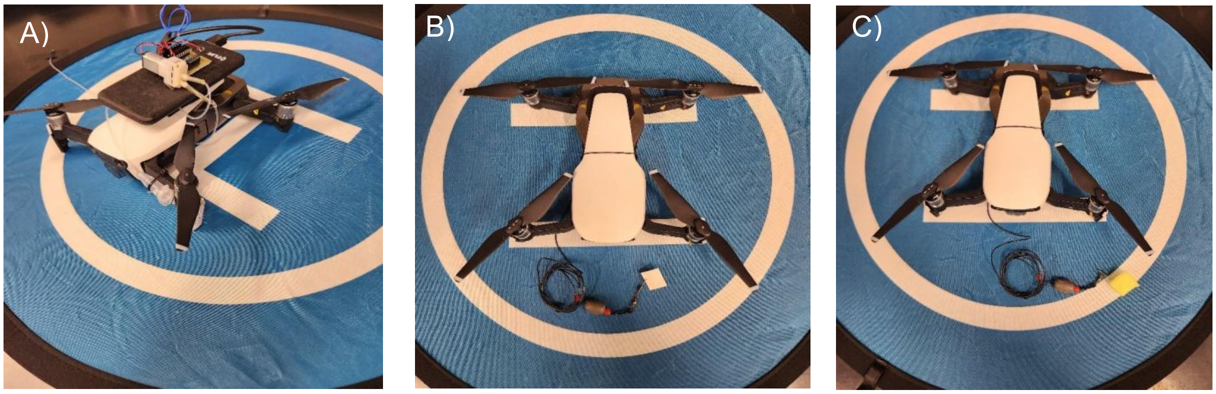

Figure S2: Pictures of the drone, adapted to perform the three different water sampling strategies. A) UAV adapted with electronic circuit to pump water using a micropump powered by a power bank. B) UAV adapted with a simple fishing line containing a paper-NADES. C) UAV adapted with a simple fishing line containing the sponge-NADES.

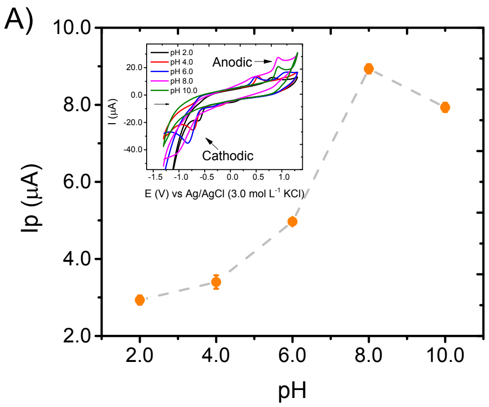

Figure S3 A: Effect of pH on the electrochemical response (Ip) of 60 µmol L^{-1} 2-NP by cyclic voltammetry (100 mV s^{-1}). All solutions were prepared in 0.5 mol L^{-1} phosphate solution with pH from 2.0 to 10.0, data collected in triplicates.

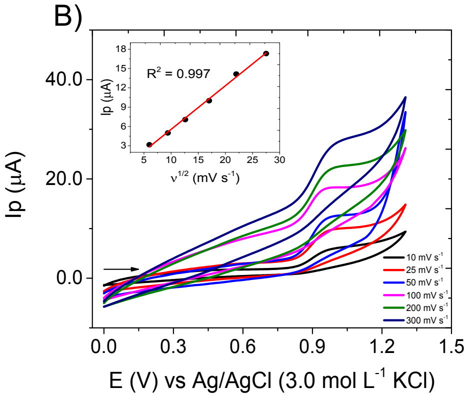

Figure S3 B: Effect of scan rates (10-500 mV/s) in the CV response of 2-NP (60 µmol L^{-1}) in 0.5 mol L^{-1} PBS (pH=8.0). Insert: dependence of the Ip vs square root of the scan rate ($v^{1/2}$).

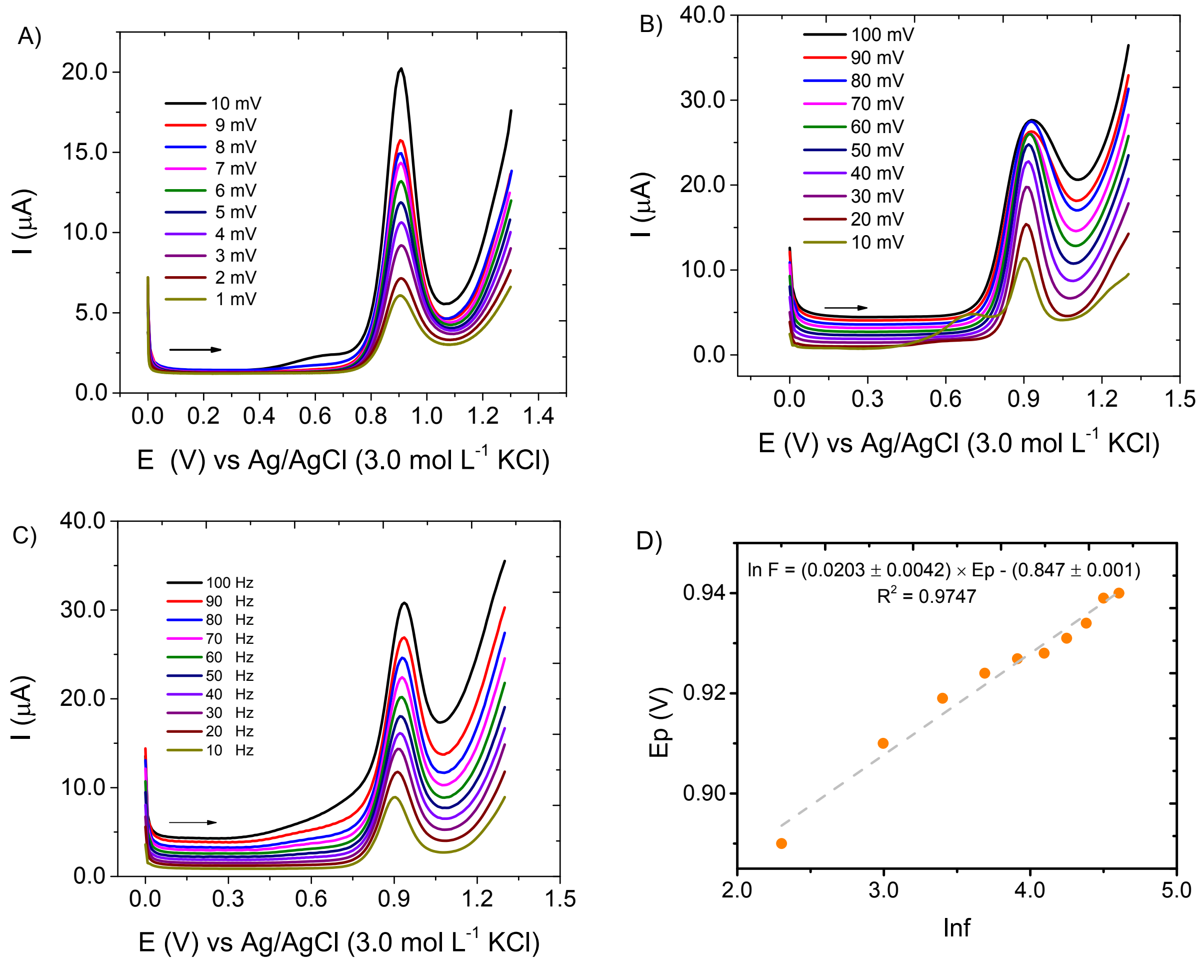

Figure S4: SWV optimization. Potential Increment study was evaluated from 1 to 10 mV with frequency of 20 Hz and amplitude of 30 mV (A). Amplitude study was ranged from 10 to 100 mV keeping constant the frequency of 20 Hz and an increment of 8 mV (B). The frequency was studied from 10 to 100 Hz with amplitude of 30 mV and an increment of 8 mV (C). For all experiments, a solution of 60.0 μmol L^{-1} of 2-NP in PBS pH 8.0 was used. Graphic of Ep vs lnf to find the number of electrons of the electrochemical oxidation of 2-NP (D).

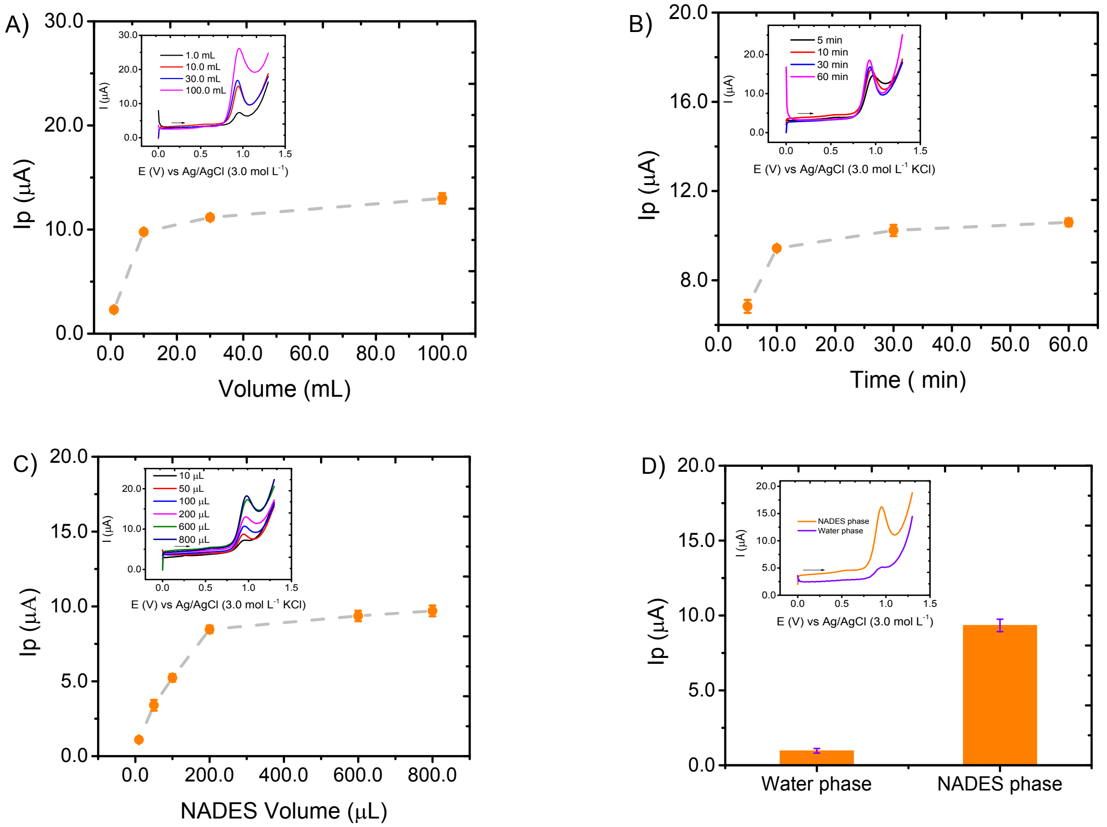

Figure S5: Results of SWV for LLE of 2-NP using NADES. Study of the volume (A) and time of extraction the solution of 2-NP (B). Evaluating of aliquot of NADES enriched with 2-NP (C) on the electrochemical oxidation of 2-NP. Comparison results of the SWV detection of 2-NP before and after LLE (D). All electrochemical experiments were performed using 30.0 mL of 30.0 µmol L^{-1} of 2-NP in PBS, pH 8.0. SWV: f = 70 Hz, ΔEs = 8 mV and a =30 mV.

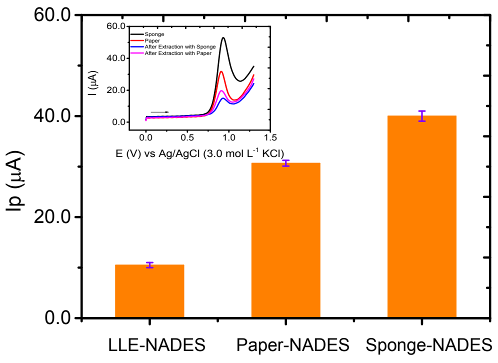

Figure S6: Results of SWV for SS-LLE of 2-NP using paper-NADES and sponge-NADES. The electroanalytical experiments were performed using 30.0 mL of 30.0 µmolL^{-1} of 2-NP aqueous solution. Electrolyte: PBS, pH 8.0. SWV: f = 70 Hz, ΔEs = 8 mV, a = 30 mV.

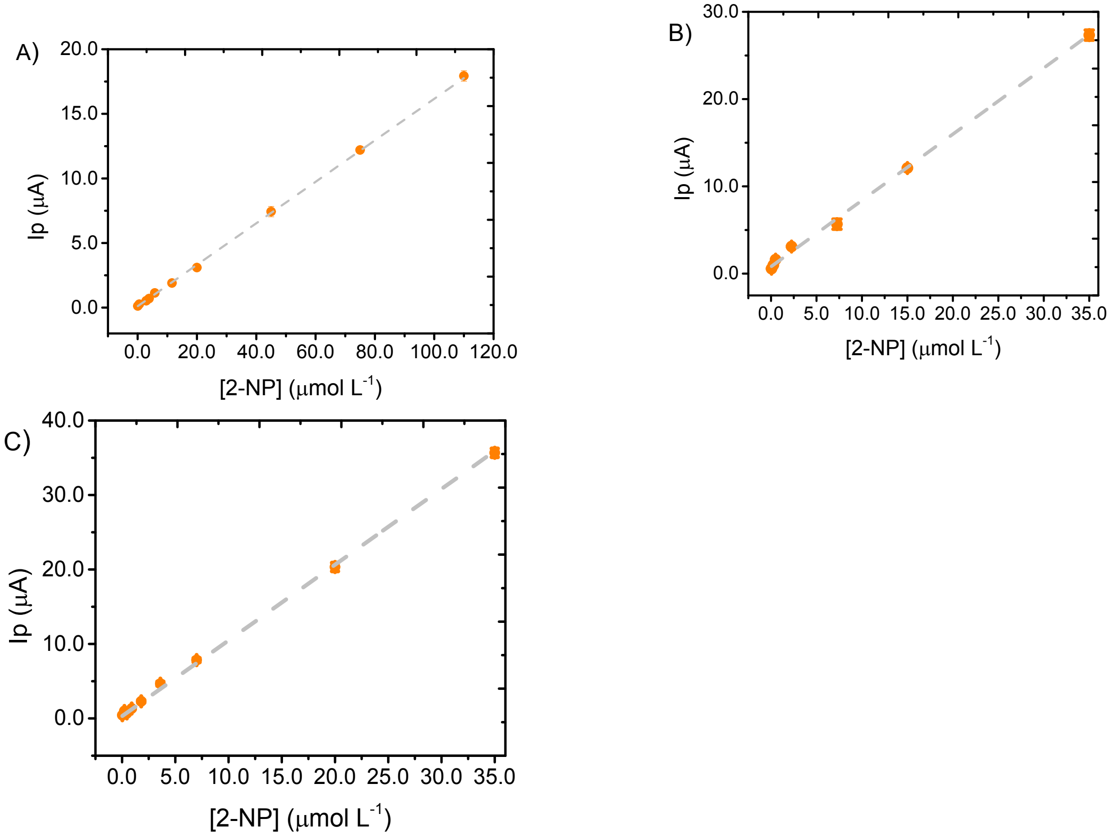

Figure S7: Analytical curves for quantification of 2-NP in water employing LLE-NADES (A), paper-NADES (SS-LLE) (B) and sponge-NADES (SS-LLE) (C), respectively. All data were obtained in triplicate. The SWVs were inserted in the analytical curves generated. SWV parameters: f = 70 Hz, ΔEs = 8 mV, a = 30 mV.

Table S1: Comparison of analytical figures of merit for various electrochemical methods reporting detection of nitrophenols.

**Electrode**	**Linear Range ($\mu mol\ L^{-1}$)**	**R^2**	**LOD ($\mu mol\ L^{-1}$)**	**Intra/Interday Variability (%)**	**Ref**
MIP[a]	2 - 400	0.996	0.2		(100)
ePADs[b]	10 - 200	0.998	1.1		(101)
Metallic gold	0.5 - 500	0.981	0.5	1.9%	(102)
Hydroxyapatite Glass Carbon	1 - 300	0.999	0.6		(103)
$La(OH)_3$-OxMWCNTs/GC[c]	1.0 - 30.0	0.997	0.27	1.7 / 2.8	(57)
CalCOP-MPC/GCE[d]	1 - 400	0.982	0.21	3.2 / 3.9	(104)
BDDE+ LLE+ NADES	0.058-110.0	0.997	0.0190	0.49 / 0.65	This work
BDDE+ Paper+ NADES	0.058-35.0	0.993	0.0039	1.31 / 3.32	This work
BDDE+ sponge+ NADES	0.025-35.0	0.992	0.0028	1.62 / 2.60	This work

[a] molecularly imprinted polymers.

[b]Electrochemical paper-based devices- Graphite pencil.

[c]lanthanum hydroxide-oxidized multi-walled carbon nanotubes/ Glass Carbon.

[d]Covalent organic polycalix[4]arenes (CalCOP)- macroporous carbon (MPC).

Table S2: Recovery data for the methodology using LLE-NADES.

Sample	Added / µmol L^{-1}	Found / µmol L^{-1}	Recovery (%)
A1	3.90	4.18 ± 0.27	107
	2.90	2.78 ± 0.27	96.0
A2	3.90	4.48 ± 0.46	115
	2.90	2.78 ± 0.47	96.0
A3	3.90	4.33 ± 0.54	111
	2.90	2.47 ± 0.26	85.4
A4	3.90	4.49 ± 0.47	115
	2.90	2.78 ± 0.26	96.0
A5	3.90	4.33 ± 0.28	111
	2.90	3.09 ± 0.46	90.7
A6	3.90	3.56 ± 0.46	91.3
	2.90	2.47 ± 0.27	85.3
A7	3.90	4.33 ± 0.27	111
	2.90	3.10 ± 0.46	106
A8	3.90	4.49 ± 0.47	115
	2.90	3.09 ± 0.47	107

Table S3: Recovery data for the methodology using paper-NADES.

Sample	Added / µmol L^{-1}	Found / µmol L^{-1}	Recovery (%)
A1	3.90	3.87 ± 0.15	99.2
	2.90	3.03 ± 0.06	105
A2	3.90	4.04 ± 0.21	103
	2.90	2.87 ± 0.07	99.0
A3	3.90	4.12 ± 0.02	106
	2.90	2.95 ± 0.05	102
A4	3.90	4.04 ± 0.08	103
	2.90	2.79 ± 0.03	96.2
A5	3.90	3.95 ± 0.05	101
	2.90	2.87 ± 0.06	99.0
A6	3.90	3.87 ± 0.07	99.2
	2.90	2.79 ± 0.08	96.2
A7	3.90	4.12 ± 0.03	106
	2.90	2.87 ± 0.07	99.0
A8	3.90	4.20 ± 0.08	108
	2.90	2.95 ± 0.04	102

Table S4: Recovery data for the methodology using sponge-NADES.

Sample	Added / µmol L^{-1}	Found / µmol L^{-1}	Recovery (%)
A1	3.90	3.61 ± 0.14	92.6
	2.90	3.01 ± 0.05	104
A2	3.90	3.86 ± 0.06	99.0
	2.90	2.89 ± 0.06	96.0
A3	3.90	3.98 ± 0.08	102
	2.90	2.76 ± 0.06	95.2
A4	3.90	3.74 ± 0.04	95.9
	2.90	3.01 ± 0.05	104
A5	3.90	4.11 ± 0.02	105
	2.90	2.89 ± 0.08	96.0
A6	3.90	3.61 ± 0.02	92.6
	2.90	3.13 ± 0.03	108
A7	3.90	3.86 ± 0.06	99.0
	2.90	2.64 ± 0.04	91.0
A8	3.90	3.98 ± 0.08	102
	2.90	3.01 ± 0.05	104
